# Passive smoking as a risk factor among older adults: an ordered probability approach for Türkiye

**DOI:** 10.3389/fpubh.2023.1142635

**Published:** 2023-06-15

**Authors:** Şeyda Ünver, Hasan Hüseyin Tekmanli, Ömer Alkan

**Affiliations:** ^1^Department of Econometrics, Faculty of Economics and Administrative Sciences, Ataturk University, Erzurum, Türkiye; ^2^Master Araştırma Eğitim ve Danışmanlık Hizmetleri Ltd. Şti., Ata Teknokent, Erzurum, Türkiye

**Keywords:** disadvantaged groups, older adults, secondhand smoke, ordered choice models, Türkiye

## Abstract

**Background/aim:**

Older adults represent a significant proportion of the population of many societies, and being one of the disadvantaged groups, they struggle with various difficulties in their social lives. Undoubtedly, passive smoking is one of these difficulties. Passive smoking among older adults, which is an important public health problem, is an issue that needs to be investigated. The aim of this study is to determine the relationship between the demographic and socio-economic characteristics of adults aged 60 and older in Türkiye and their exposure to secondhand smoke (SHS).

**Methods:**

In this study, a microdata set of the 2016 and 2019 Turkey Health Survey conducted by the Turkish Statistical Institute (TUIK) was used. This survey was conducted by TUIK in the relevant years using a stratified sampling method to best represent the whole of Turkey. The study considered only demographic and socio-economic characteristics to investigate passive smoking. Since all the variables used in the study were categorical, the relationship between the dependent variable and the independent variables was first investigated using chi-square tests. In addition, since the dependent variable has an ordered-categorical probability form, the generalized ordinal logit model was used for the analysis of passive smoking and related factors.

**Results:**

The rate of exposure to tobacco smoke of older adults who participated in the study in 2016 was 16%, while the rate of those who participated in the study in 2019 was 21%.

**Conclusion:**

According to the findings of the study, older, uneducated, and uninsured smokers carry a more serious SHS risk. It may be beneficial for society for policy makers to carry out studies by considering these features a priority and focusing on policies in this context. Expanding smoke-free areas to cover older adult, increasing penalties as a deterrent, facilitating education, increasing state support for education, increasing education and public service announcements about tobacco harms, and facilitating social security are the main examples. This study’s findings are crucial as a source of information for the development of policies and programs aimed at preventing the exposure of older adults to tobacco smoke.

## 1. Introduction

Tobacco usage is a severe threat to global health (1). More than 8 million individuals die annually from tobacco usage, and more than 7 million of these deaths are caused by direct tobacco use, while approximately 1.2 million are the result of secondhand smoke (SHS) (2). While active smoking is a voluntary behavior, SHS exposure occurs passively and can also affect non-smokers (3).

SHS exposure, which poses significant threats to human health, is the third leading cause of preventable deaths in the world (4). Similar to active smoking, passive smoking is a severe risk factor for many health morbidities (5). Various studies have found a relationship between SHS and cognitive impairment, dementia, and other neurodegenerative diseases (6–8). Exposure to SHS not only results in serious illness, but also imposes a significant economic and societal burden (3). SHS imposes direct and indirect expenses on national economies in addition to its ever-increasing health burden. Each year, billions of dollars are spent owing to this health issue (9). A review of the relevant literature has been examined, and it has been discovered that demographic and socio-economic factors significantly affect SHS. It has also been identified in previous studies that SHS is potentially associated with adverse health effects (9–11).

Exposure to tobacco smoke is an important public health problem. In addition, it has been discussed in the scientific world with different methods and perspectives for decades. The topic of infant and adolescent tobacco smoke exposure has also been investigated (12–15). Studies that make no distinction based on age are also relatively available (16–19), but there are very few adult-focused studies (20). Some studies have also addressed women’s exposure to tobacco smoke (21–25). However, there is a very serious gap in the exposure of older adults to tobacco smoke. Undoubtedly, older adults are also one of the disadvantaged groups.

Aging is a physiological process that results in death and is affected by genetic factors, lifestyle choices, and environmental exposures. The effects of aging, which include changes in health requirements, last a long time in a person’s life (26). The global population is rapidly aging, particularly in low- and middle-income countries (27). The older population (those aged 60 and over) is expanding more quickly than the general population worldwide. This situation has been attributed to increasing life expectancy and decreasing birth rates (28). Globally, the proportion of older adults (60 years and older) is expected to reach 21% by 2050 (29). Successful aging has developed into a critical term for defining the standard of aging. The definition evolved from a biomedical perspective to include a broader view of late-life cultural and mental adjustment mechanisms (30).

SHS exposure among older adults is a significant and potentially preventable public health issue (31). On the other hand, mental health entails a positive relationship with people and the pursuit of a productive and fulfilling existence (32). Due to age-related physiological changes and the development of severe health conditions, older adults may be particularly sensitive to the effects of SHS. In addition, older adults may be at a higher risk of involuntary exposure to SHS. This is because these people spend most of their time indoors and are at a greater risk of economic dependency (33). Various studies have found that exposure to SHS is a well-known risk factor for cardiovascular disorders (such as stroke, angina, and hypertension) and lung cancer in adults who do not smoke (6, 34–37). However, few studies have examined the effects of SHS exposure on the health of older adults (33).

In a study conducted in Italy, 33% of the older population was found to be exposed to SHS indoors (38). Another study conducted among nonsmokers over 60 years of age that aimed to evaluate the relationship between SHS and frailty syndrome in older nonsmokers found that exposure to SHS was associated with an increased frequency of frailty (33). In addition, it was stated in a study that the mental health and suicidal tendencies of older adults attracted a lot of attention due to their fragility. As a result of the study, it was determined that older men who had sex with men had higher depression and suicidality scores (39). Passive smoking was found to be significantly associated with an increased prevalence of hypertension and a lower control rate in the older population in a study conducted in Beijing, China (40). As a result of a study conducted to investigate the effects of passive smoking exposure on cognitive function in older adults, it was determined that passive smoking exposure is associated with an increased risk of cognitive impairment (6).

The concept of “old age” encompasses various dimensions, including chronological, biological, psychological, and social age. The United Nations defines “older adults” as those aged 60 and above (41). Previous studies have categorized individuals in this age group as “older adults” but little is known about their exposure to tobacco smoke in Türkiye (6, 28, 29, 31, 33, 42). This study aims to fill this gap by identifying factors associated with tobacco exposure in adults aged 60 and above in Türkiye, which can help identify those at higher risk. Overall, the language could be made more concise and easier to follow with clearer phrasing.

## 2. Methods

### 2.1. Data

In this study, a microdata set obtained from the Turkey Health Survey conducted by TURKSTAT in 2016 and 2019 was used. The Turkey Health Survey was conducted for the first time in 2008. This research has been applied to households by the face-to-face interview method, every 2 years until 2016, and every 3 years as of 2019. The latest data for 2019 have been shared by TURKSTAT (43, 44).

All cities, towns, and villages within the borders of the Republic of Türkiye were included in the populations for the stratified two-stage cluster sampling method used to obtain data. Rural–urban distinction was used as an external stratifying criterion (areas with population under and equal to 20,000 were considered rural, while areas with population equal to and above 20,001 were considered urban). The first-stage sampling unit comprised blocks selected from groups (blocks) consisting of an average of 100 house addresses, and the second-stage sampling unit consisted of systematically selected households from each selected group. Cross-sectional data on individuals above the age of 15 were used in this study for sample selection (43, 44). The scope of the study included older adults over 60 years of age in Turkey (45). There are 3,657 observations for 2016 and 3,595 observations for 2019 in the data set.

### 2.2. Outcome variables

The dependent variable of the study was the exposure of older adults to tobacco smoke. The dependent variable of the model consists of three categories in terms of the frequency of exposure to tobacco: exposure more than 1 hour per day, exposure less than 1 hour per day, and no exposure at all.

### 2.3. Independent variables

The independent variables included in the study were those from the Turkey Health Survey. The independent variables of the study are as follows: year (2016, 2019), gender (male, female), age (60–64, 65 and older), ability to afford treatment (yes, no), education (illiterate or did not complete primary school, primary school, high school, university), health problems (yes, no), tobacco use (yes, no), and reliable relatives (none, 1–2 people, 3 or more people).

Ordinal and nominal variables were defined as dummy variables so that the effects of the categories of all variables included in the binary logistic regression model could be observed (46, 47).

### 2.4. Analysis method

The statistical analysis in this study utilized Stata 15 software (Stata Corporation) to account for the complex sampling design and weighting. To obtain a more accurate representation of the population, a weighted analysis was conducted, as described by Coşkun et al. (48). First, the tobacco exposure status of the research participants was determined, along with the frequency and proportions of the independent variables. Subsequently, a generalized ordered logistic regression model was employed to investigate the factors associated with tobacco exposure among older adults.

## 3. Results

### 3.1. Descriptive statistics and crosstabs

Tables 1, 2 provide descriptive statistics and chi-square independence test results for the variables used in the study. According to the descriptive statistics of the study, the rate of exposure to tobacco smoke of older adults who participated in the study in 2016 was 16%, while the rate of those who participated in the study in 2019 was 21%.

**Table 1:** Findings on the factors affecting the exposure of older adults to tobacco smoke for 2016 (*n* = 3,657).

Variables	*n* (%)	Tobacco exposure (%)			*p*
		Exposure more than one hour per day	Exposure less than one hour per day	No exposure at all	
Gender					
Male	1,617 (44.22)	8.09	11.04	80.87	0.004^a^
Female	2,040 (55.78)	5.82	8.91	85.27	
Age					
60–64	1,145 (31.31)	8.81	12.27	78.92	0.000^a^
65+	2,512 (68.69)	5.87	8.68	85.46	
Education					
Illiterate	1,487 (40.66)	6.51	9.62	83.87	0.398
Primary school	1,591 (41.54)	7.57	9.5	82.93	
Middle school	209 (5.72)	4.16	12.77	83.07	
High school	215 (5.88)	8.15	13.65	78.19	
University	227 (6.21)	5.34	7.8	86.86	
Are your treatment costs covered by the social security institution?					
No	433 (11.84)	10.98	10.68	78.34	0.000^a^
Yes	3,224 (88.16)	6.27	9.77	83.96	
Have you ever been unable to make health expenses due to payment difficulties?					
No	3,329 (91.03)	6.39	9.86	83.75	0.017^b^
Yes	328 (8.97)	11.36	10.06	78.59	
Do you smoke?					
No	3,145 (86)	3.8	8.07	88.13	0.000^a^
Yes	512 (14)	24.32	20.23	55.45	
How many people close to you can you trust if you have a serious personal problem?					
None	219 (5.99)	6.09	8.14	85.77	0.126
1**–**2	1,453 (39.73)	7.72	10.31	81.97	
3+	1,985 (54.28)	6.29	9.76	83.96	

a *p* < 0.01.

b *p* < 0.05.

**Table 2:** Findings on the factors affecting the exposure of older adults to tobacco smoke for 2019 (*n* = 3,595).

Variables	*n* (%)	Tobacco exposure (%)			*p*
		Exposure more than one hour per day	Exposure less than one hour per day	No exposure at all	
Gender					
Male	1,633 (45.42)	10.2	4.78	85.02	0.000^a^
Female	1,962 (54.58)	6.53	3.13	90.34	
Age					
60–64	1,137 (31.63)	10.65	4.74	84.61	0.000^a^
65+	2,458 (68.37)	7.09	3.49	89.42	
Education					
Illiterate	1,211 (33.69)	8.11	2.59	8.93	0.025^b^
Primary school	1,642 (45.67)	7.5	4.78	87.71	
Middle school	190 (5.29)	10.15	1.71	88.13	
High school	289 (8.04)	10.6	5.96	83.44	
University	263 (7.32)	9.5	4.42	86.08	
Are your treatment costs covered by the social security institution?					
No	89 (2.48)	7.75	1.75	90.5	0.473
Yes	3,506 (97.52)	8.24	3.95	87.82	
Have you ever been unable to make health expenses due to payment difficulties?					
No	3,282 (91.29)	7.88	3.67	88.46	0.000^a^
Yes	313 (8.71)	11.89	6.2	81.9	
Do you smoke?					
No	2,984 (83)	4.22	3.3	92.48	0.000^a^
Yes	611 (17)	28.77	6.91	64.32	
How many people close to you can you trust if you have a serious personal problem?					
None	1,96 (5.45)	7.76	4.66	87.57	0.384
1**–**2	1,366 (38)	8.84	4.26	56.9	
3+	2,033 (56.55)	7.85	3.55	8.86	

a *p* < 0.01.

b *p* < 0.05.

Table 1 presents the findings on the factors affecting the exposure of older adults to tobacco smoke for 2016. The data includes the number and percentage of research participants based on their tobacco exposure status and various independent variables.

The *p*-values in the table show the significance of the association between each independent variable and tobacco exposure. A, b, and c show the statistical significance of the coefficients at the 1, 5, and 10% significance levels, respectively. For example, a *p*-value of less than 0.05 (indicated by “b”) indicates a statistically significant association.

According to the significant findings, males have significantly higher tobacco exposure than females. The research participants aged 60–64 have significantly higher tobacco exposure than those aged 65 and above. The participants whose treatment costs are covered by the social security institution have significantly higher tobacco exposure than those who are not. The research participants who have faced payment difficulties have significantly higher tobacco exposure than those who have not. Smokers have significantly higher tobacco exposure than non-smokers.

Table 2 presents the findings on the factors affecting the exposure of older adults to tobacco smoke for 2019. According to the significant findings; males have significantly higher tobacco exposure than females. The research participants aged 60–64 have significantly higher tobacco exposure than those aged 65 and above. Illiterate participants have significantly lower tobacco exposure than those with primary education. The research participants who have faced payment difficulties have significantly higher tobacco exposure than those who have not. Smokers have a significantly higher tobacco exposure than non-smokers.

### 3.2. Econometric estimation

As with the independent variables, the generalized ordered logit model generates separate equations for each remaining category by using one category of the dependent variable as a reference (49, 50). Here, the category “no exposure” is mentioned. Tables 3, 4 provide the estimation results and marginal effects of the model. The fact that all the variance inflation factor values related to the independent variables are less than 5 indicates that there is no multicollinearity problem (51, 52). Tables 3, 4 display the model estimation results and marginal effects. The marginal effects will be interpreted for the exposure categories “more than 1 hour per day” and “less than 1 hour per day” respectively.

**Table 3:** Estimated model results and marginal effects of factors associated with older adults’ exposure to tobacco smoke for 2016.

Variables	Exposure more than one hour per day				Exposure less than one hour per day				1/VIF
	COR	AOR	dy/dx	Std. Err.	COR	AOR	dy/dx	Std. Err.	
Gender (Ref: Male)									
Female	0.846	0.688^c^	0.021^c^	0.011	0.971	0.921	−0.011	0.014	0.803
Age (Ref: 60–64)									
65+	1.308^c^	1.296	−0.015	0.011	1.372^a^	1.427^a^	−0.031^b^	0.014	0.941
Education (Ref: Illiterate)									
Primary school	1.025	0.928	0.004	0.014	1.304^b^	1.196	−0.027^c^	0.016	0.695
Middle school	1.585	2.379^b^	−0.037^b^	0.015	1.201	1.374	−0.001	0.025	0.859
High school	1.066	0.999	6^*^10^−5^	0.021	1.312	1.019	−0.003	0.031	0.836
University	2.164^c^	1.596	−0.023	0.021	1.881^a^	1.928^b^	−0.049^b^	0.02	0.827
Are your treatment costs covered by the social security institution? (Ref: No)									
Yes	1.962^a^	1.800^b^	−0.039^b^	0.0184	1.361^b^	1.453^b^	−0.011	0.023	0.908
Have you ever been unable to make health expenses due to payment difficulties? (Ref: No)									
Yes	0.696	0.711	0.021	0.019	0.903	0.846	0.0001	0.022	0.953
Do you smoke? (Ref: No)									
Yes	0.133^a^	0.099^a^	0.233^a^	0.029	0.149^a^	0.154^a^	0.105^a^	0.025	0.914
How many people close to you can you trust if you have a serious personal problem? (Ref: None)									
1–2	0.804	0.497^b^	0.036^b^	0.015	0.743	0.571^b^	0.027	0.021	0.216
3+	1.094	0.654	0.019	0.014	0.957	0.649^c^	0.027	0.021	0.213
Constant	14.395^a^	28.879^a^			5.669^a^	6.509^a^			

a *p* < 0.01.

b *p* < 0.05.

c *p* < 0.10.

COR, Crude Odds Ratio; AOR, Adjusted Odds Ratio.

**Table 4:** Estimated model results and marginal effects of factors associated with older adults’ exposure to tobacco smoke for 2019.

Variables	Exposure more than one hour per day				Exposure less than one hour per day				1/VIF
	COR	AOR	dy/dx	Std. Err.	COR	AOR	dy/dx	Std. Err.	
Gender (Ref: Male)									
Female	1.015	0.998	0.0001	0.009	1.175	1.155	−0.014^c^	0.008	0.825
Age (Ref: 60–64)									
65+	1.162	1.109	−0.007	0.010	1.290^b^	1.206	−0.011	0.008	0.940
Education (Ref: Illiterate)									
Primary school	1.532^a^	1.657^a^	−0.035^a^	0.013	1.220	1.263	0.013	0.008	0.680
Middle school	1.278	1.402	−0.025	0.020	1.485	1.653^c^	−0.021^b^	0.008	0.853
High school	1.685^b^	1.673^c^	−0.036^b^	0.018	1.356	1.370	0.006	0.013	0.773
University	1.761^b^	1.835^b^	−0.041^b^	0.016	1.284	1.309	0.015	0.012	0.805
Are your treatment costs covered by the social security institution? (Ref: No)									
Yes	0.721	1.016	−0.001	0.033	0.706	0.834	0.017	0.016	0.979
Have you ever been unable to make health expenses due to payment difficulties? (Ref: No)									
Yes	0.652^b^	0.677^c^	0.029	0.019	0.539^a^	0.610^b^	0.024^c^	0.014	0.979
Do you smoke? (Ref: No)									
Yes	0.110^a^	0.098^a^	0.262^a^	0.024	0.149^a^	0.147^a^	0.015	0.012	0.907
How many people close to you can you trust if you have a serious personal problem? (Ref: None)									
1–2	0.657	0.709	0.022	0.019	0.767	0.787	0.001	0.014	0.201
3+	0.832	0.794	0.014	0.018	0.922	0.883	−0.003	0.013	0.199
Constant	28.368^a^	21.299^a^			14.682^a^	12.591^a^			

a *p* < 0.01.

b *p* < 0.05.

c *p* < 0.10.

COR, Crude Odds Ratio; AOR, Adjusted Odds Ratio.

According to Table 3, women are 2.1% more likely than men to be exposed to tobacco smoke for more than 1 hour a day. Compared to individuals aged 60 to 64, those aged 65 and over are 3.1% less likely to be exposed to tobacco smoke for less than 1 hour a day. Primary school graduates are 2.7% less likely than illiterates to be exposed to tobacco smoke for less than 1 hour a day. Elementary school graduates are 3.7% less likely than illiterates to be exposed to tobacco smoke for more than 1 hour a day. University graduates are 4.9% less likely than illiterates to be exposed to tobacco smoke for less than 1 hour a day. Those whose treatment expenses are paid by a social security institution are 3.9% less likely to be exposed to tobacco smoke for more than 1 hour a day. Tobacco smokers are 23.3% more likely than nonsmokers to be exposed to tobacco smoke for more than an hour a day. Tobacco users are 10.5% more likely than nonsmokers to be exposed to tobacco smoke for less than 1 hour a day. Individuals who can rely on one or two people in an emergency are 3.6% more likely to be exposed to tobacco smoke for more than an hour a day compared to those who cannot.

Table 4 indicates that women are 1.4% less likely than men to be exposed to tobacco smoke for less than 1 hour a day. Graduates of primary school are 3.5% less likely than illiterates to be exposed to tobacco smoke for more than an hour every day. Graduates of elementary school are 2.1% less likely than illiterates to be exposed to tobacco smoke for less than an hour every day. Graduates of high school are 3.6% less likely than illiterates to be exposed to tobacco smoke for more than an hour a day. University graduates are 4.1% less likely than illiterates to be exposed to tobacco smoke for more than 1 hour a day. Individuals with problems affording health care are 2.4% more likely to be exposed to cigarette smoke for less than an hour each day than those without such difficulties. Nonsmokers are 26.2% less likely than smokers to be exposed to tobacco smoke for more than 1 hour every day.

## 4. Discussion

According to the descriptive statistics of the study, the rate of exposure to tobacco smoke of older adults who participated in the study in 2016 was 16%, while the rate of those who participated in the study in 2019 was 21%. This rate is quite low compared to other countries. According to a study conducted in Egypt, 37.1% of older adults were exposed to tobacco smoke (53). In a study conducted in China, the exposure rate for those aged 40 and older to tobacco smoke was found to be 21.35% (54), and in another study, it was found to be 24.8% for those aged 55 and older (31). In a study conducted in the United States, the proportion of older adults exposed to tobacco smoke was found to be 25.2% (55). According to a study conducted in eight sub-Saharan countries, the rates of exposure to tobacco smoke among those aged 65 and older are 20% in Cameroon, 17.5% in Kenya, 17.2% in Uganda, 9.5% in Nigeria, 18% in Senegal, 4% in Ethiopia, 21% in Botswana, and 13.5% in Tanzania (56).

According to a study conducted in Beijing, 40.5 and 41.1% of women aged 60–70 were exposed to tobacco smoke in 2001 and 2010, respectively. In the aforementioned study, it was determined that the prevalence of passive smoking was much lower among individuals with higher levels of education. In addition, according to the study, intense physical activity was associated with the greatest increase in the prevalence of passive smoking among males (15.2%), whereas light labor was associated with the highest decrease (7.3%) among women (31). In another study conducted on Chinese women, the rate of exposure to tobacco smoke was found to be 32.51% among women between the ages of 30 and 40 and 17.77% among women older than 40 (54). In a study conducted on individuals over the age of 60 in Zhejiang province, it was determined that the rate of exposure to tobacco smoke was 15.4% among married individuals and 16.7% among unmarried individuals. In the aforementioned study, it was determined that nonworking individuals were exposed to tobacco smoke at a rate of 19.2%, while working individuals were exposed at a rate of 15.5%. In addition, the rate of exposure to tobacco smoke among alcohol consumers in the study was reported to be 13.9% (6).

According to the findings of this study, women are more likely than men to be exposed to tobacco smoke. Similarly, according to a study conducted in India, rural women are more likely than men to be exposed to tobacco smoke (57). In a study conducted in Northern China, it was found that women were less likely to be exposed to tobacco smoke in cities (58). The findings of the study indicated that those aged 65 and older were less likely to be exposed to tobacco smoke than those aged 60 to 64. In a study conducted on women in Türkiye, it was determined that other age groups were more likely to be exposed to tobacco smoke than those aged 65 and older (21). Again, in a study examining the likelihood of exposure to tobacco smoke by education level in Türkiye, it was determined that those aged 55 and older were less likely to be exposed to tobacco smoke than younger age groups (4). In a study for Greece, age groups 25–39, 40–54, and 55–64 were found to be more likely to be exposed to tobacco smoke than age group 65+ (59). Similarly, according to the findings of a study conducted in China, tobacco smoke exposure decreases with age (60). In a study conducted in the United States, it was determined that the age groups 40–59 and 60+ were less likely to be exposed to tobacco smoke than the age group 20–39 (61). This group’s withdrawal from the workforce may be a contributing factor to the low rate and probability of tobacco smoke exposure among older adults. In fact, a study conducted in China found that retirees were less likely to be exposed to tobacco smoke, supporting our finding because these individuals are no longer employed and are not frequently in places where tobacco is used (31). The decreasing possibilities with increasing age show the necessity of focusing on the youth in the policies to be followed. In addition to tobacco bans, it may be advantageous to emphasize tobacco’s negative effects more frequently in educational programs. Furthermore, more emphasis on healthy lives in public service announcements, TV, and radio programs may also be effective.

Graduates of primary, middle, high school, and university are less likely to be exposed to tobacco smoke than illiterates. Higher education level refers to higher income levels at both state and private institutions, and individuals are more conscious of their health (62). A study conducted on different education levels in Türkiye, it was determined that those with a higher level of education were less likely to be exposed to tobacco smoke than illiterates (4). Similar to this study’s findings, studies conducted in China, India, and Bangladesh found that exposure to tobacco smoke increased with decreasing levels of education (60, 63, 64). This demonstrates that the uneducated population faces greater risk and underlines the necessity of targeted education programs. Educating tobacco users about smoke-free homes and the dangers of tobacco is an essential element in reversing this situation and can reduce tobacco use and exposure (65). In contrast to the findings of this study, a study conducted in the United States found that those with a higher level of education were more exposed to tobacco smoke than those with a lower level of education (61).

Those whose medical expenses are covered by the social security institution are less likely to be exposed to SHS. In a study conducted in Türkiye, it was determined that men whose medical expenses were covered by social security institutions were less likely to use tobacco (66). Today, the use of preventive health services in the fight against health problems is becoming common. With preventive health services, individuals can be protected against diseases. They also reduce health care costs and prevent workforce loss. Unquestionably, preventive health services are crucial for reducing tobacco use and exposure, which is an important public health problem (67).

Tobacco users are more likely than others to be exposed to tobacco smoke. Studies conducted in the United States and India have also found that tobacco users are more likely to be exposed to tobacco smoke (64, 68). The low exposure probability of non-smokers is an expected outcome. This may highlight the need for population-based education campaigns that provide smoking cessation advice or information on how to assist a smoking friend or family member in quitting. Such campaigns may also be opportunities to encourage nonsmokers to become active participants in the enforcement of the smoke-free law, for instance, by contacting the relevant authorities when the indoor smoking ban is violated. Such concerted efforts by nonsmoking individuals and law enforcement can help normalize smoking in public places and promote smoke-free environments (16).

Those who have a number of close contacts to trust in the event of a serious personal problem are more likely than those who have no exposure to tobacco smoke. In a study conducted on infants in Türkiye, extended families were found to have a higher risk of exposure to tobacco smoke (69).

## 5. Strengths and limitations of this study

This study was conducted using data obtained from the official statistical institution of Türkiye, TurkStat. To the best of our knowledge, this is the first known study to determine the factors associated with exposure to tobacco smoke in adults aged 60 and older in Türkiye. The study’s findings are applicable to Turkish society in 2016 and 2019, but cannot be generalized to other years or societies. The aim of this study is to shed light on the policies to be developed by competent authorities. In the future, multivariate probability approaches, in which infants or adolescents, who are also disadvantaged segments of society, can be discussed together with older adults to obtain more detailed findings.

This study is not without limitations. To begin, the study relies on secondary data. The variables required for statistical analysis are those found in the data-set (70). Additionally, some variables, such as individuals’ occupations, home ownership status, and levels of exposure to tobacco smoke by parents, siblings, and other household members and friends were not included in the analysis. Because the data are cross-sectional, the definite causal relationship between verbal violations and related socio-economic factors cannot be inferred (71). Furthermore, because the dataset did not include information about the location of tobacco smoke exposure, this study focused on general SHS exposure. The distinction between SHS-exposed locations, such as homes, public places, workplaces, restaurants, and bars, was omitted. Because tests to determine individuals’ exposure to tobacco smoke could not be conducted in a laboratory setting, the study relied on individuals’ own responses. The data obtained might be biased as a result of this data collection method.

## 6. Conclusion

Tobacco use causes significant health problems not only for tobacco users, but also for people who do not use tobacco, due to environmental tobacco smoke spreading to the environment. Each year, new evidence emerges regarding the health risks associated with tobacco smoke exposure. To reduce the prevalence of SHS, it is essential to understand the factors that determine tobacco use.

In this study, the data of older adults who participated in the 2016 and 2019 Turkey Health Survey conducted by the Turkish Statistical Institute were used. In this study, using a generalized ordinal logistic regression model, factors related to the exposure of older adults to tobacco smoke in Türkiye were identified.

Tobacco smoke exposure results in disease, death, loss of the workforce, etc. for society. In recent years, the Turkish government has taken serious measures to combat the damage caused by tobacco. However, the majority of the measures taken are for public areas, and there is little intervention in homes where older adults spend the majority of their time. The research shows that smoke-free laws have a positive relationship with smoke-free house rules. This suggests that prohibiting smoking in public places could have far-reaching effects on reducing exposure to SHS in other settings (72). The public should be made aware of the dangers of SHS, particularly in private areas, and awareness studies should be conducted. Through channels such as social media, the positive effects of a smoke-free home environment on a healthy life should frequently be publicized (73). It has been shown that comprehensive smoke-free laws are significantly associated with fewer hospitalizations, deaths from coronary events, and other heart diseases (74). Therefore, strengthening SHS policies will reduce deaths from SHS exposure and the associated economic burden (75). In addition, smoking cessation efforts are a cornerstone in the prevention of tobacco harm, and this issue should be addressed separately as part of organized tobacco-control efforts (76).

Therefore, it is assumed that the sample in question best represents society. The study’s findings are applicable to Turkish society in 2016 and 2019, but cannot be generalized to other years or societies. The aim of this study is to shed light on the policies to be developed by competent authorities. In the future, multivariate probability approaches in which infants or adolescents, who are also disadvantaged segments of society, can be discussed together with older adults to obtain more detailed findings.

## Data availability statement

The data analyzed in this study is subject to the following licenses/restrictions: The data underlying this study is subject to third-party restrictions by the Turkey Statistical Institute. Data are available from the Turkish Statistical Institute (bilgi@tuik.gov.tr) for researchers who meet the criteria for access to confidential data. The authors of the study did not receive any special privileges in accessing the data. Requests to access these datasets should be directed to bilgi@tuik.gov.tr.

## Author contributions

ÖA: conceptualization, methodology, software, data curation, writing - original draft, writing-review and editing, formal analysis, and visualization. HT: conceptualization, methodology, formal analysis, data curation, supervision, writing- original draft, and editing. ŞÜ: conceptualization, supervision, and editing. All authors contributed to the article and approved the submitted version.

## Conflict of interest

ÖA was employed by Master Araştırma Eğitim ve Danışmanlık Hizmetleri Ltd. Şti., Ata Teknokent.

The remaining authors declare that the research was conducted in the absence of any commercial or financial relationships that could be construed as a potential conflict of interest.

The reviewer EO declared a shared affiliation with the author(s) to the handling editor at the time of review.

## Publisher’s note

All claims expressed in this article are solely those of the authors and do not necessarily represent those of their affiliated organizations, or those of the publisher, the editors and the reviewers. Any product that may be evaluated in this article, or claim that may be made by its manufacturer, is not guaranteed or endorsed by the publisher.

## Author disclaimer

The views and opinions expressed in this manuscript are those of the authors only and do not necessarily represent the views, official policy, or position of the Turkish Statistical Institute.
